# Actin filament reorganisation controlled by the SCAR/WAVE complex mediates stomatal response to darkness

**DOI:** 10.1111/nph.14655

**Published:** 2017-06-21

**Authors:** Jean‐Charles Isner, Zaoxu Xu, Joaquim Miguel Costa, Fabien Monnet, Thomas Batstone, Xiaobin Ou, Michael J. Deeks, Bernard Genty, Kun Jiang, Alistair M. Hetherington

**Affiliations:** ^1^ School of Biological Sciences University of Bristol Life Sciences Building, 24 Tyndall Avenue Bristol BS8 1TQ UK; ^2^ College of Life Sciences Zhejiang University Hangzhou Zhejiang Province 310058 China; ^3^ Commissariat à l'Energie Atomique et aux Energies Alternatives Centre National de la Recherche Scientifique UMR 7265 Université Aix‐Marseille, Biologie Végétale et Microbiologie Environnementales Saint‐Paul‐lez‐Durance 13108 France; ^4^ Department of Biosciences University of Exeter Exeter EX4 4QD UK

**Keywords:** actin filaments, Arp2/3 complex, darkness, SCAR/WAVE complex, Stomata

## Abstract

- Stomata respond to darkness by closing to prevent excessive water loss during the night. Although the reorganisation of actin filaments during stomatal closure is documented, the underlying mechanisms responsible for dark‐induced cytoskeletal arrangement remain largely unknown.
- We used genetic, physiological and cell biological approaches to show that reorganisation of the actin cytoskeleton is required for dark‐induced stomatal closure.
- The *opal5* mutant does not close in response to darkness but exhibits wild‐type (WT) behaviour when exposed to abscisic acid (ABA) or CaCl_2_. The mutation was mapped to At5g18410, encoding the PIR/SRA1/KLK subunit of the *Arabidopsis* SCAR/WAVE complex. Stomata of an independent allele of the *PIR* gene (*Atpir‐1*) showed reduced sensitivity to darkness and F_1_ progenies of the cross between *opal5* and *Atpir‐1* displayed distorted leaf trichomes, suggesting that the two mutants are allelic. Darkness induced changes in the extent of actin filament bundling in WT. These were abolished in *opal5*. Disruption of filamentous actin using latrunculin B or cytochalasin D restored wild‐type stomatal sensitivity to darkness in *opal5*.
- Our findings suggest that the stomatal response to darkness is mediated by reorganisation of guard cell actin filaments, a process that is finely tuned by the conserved SCAR/WAVE–Arp2/3 actin regulatory module.

## Introduction

Stomata are pores found predominantly on the leaf surfaces that regulate gas exchange between plants and the environment. Pairs of guard cells that enclose the stomatal pore perceive and respond to changes in environmental signals such that the aperture of the pore changes (Hetherington & Woodward, 2003). Stomatal opening is stimulated by blue and red light via distinct signalling mechanisms (Kinoshita & Shimazaki, 1999, 2002; Kinoshita *et al*., 2001; Wang *et al*., 2010), and involves catabolism of starch and lipid in guard cells (Horrer *et al*., 2016; McLachlan *et al*., 2016). In contrast to the significant progress made towards the understanding of light‐promoted stomatal opening (Shimazaki *et al*., 2007) we know much less about the cellular processes behind dark‐induced stomatal closure.

Several *Arabidopsis* mutants deficient in the stomatal response to darkness have been identified in the past decade. These were found to affect either photomorphogenesis (Liang *et al*., 2005; Mao *et al*., 2005) or regulation of ion channels and transporters (Merlot *et al*., 2007; Negi *et al*., 2008; Vahisalu *et al*., 2008). These mutations also cause pleiotropic developmental defects, which prevent their utilisation in studies of the impact of nighttime transpiration (*E*_night_) on plant growth and water use efficiency (Caird *et al*., 2007). A recent genetic screen recovered five stomatal mutants (*opal1–5*) that display reduced sensitivity to darkness but no discernible lesions in leaf morphology or in plant growth (Costa *et al*., 2015). It is likely that further characterisation of these *opal* mutants will provide more information on guard cell dark‐induced signalling and contribute further to our understanding of the impact of *E*_night_ on plant fitness (Coupel‐Ledru *et al*., 2016).

There is a growing body of evidence suggesting that fast reconfiguration of guard‐cell cortical actin bundles occurs during stomatal closure triggered by darkness, abscisic acid (ABA) and pathogen‐associated molecular patterns (Eun & Lee, 1997; Gao *et al*., 2008; Shimono *et al*., 2016). Moreover, stabilisation of filamentous actin using phalloidin prevents ABA‐induced stomatal closure, indicating an important function of actin reorganisation in stomatal regulation (Kim *et al*., 1995). Further insight into the regulatory role of actin dynamics in stomatal movement was provided by patch‐clamp analysis of stretch‐activated calcium‐permeable channels in the plasma membrane of *Vicia faba* guard cells (Zhang *et al*., 2007). It was shown that actin disassembly facilitates the stretch activation of these channels, and subsequently mediates elevation of cytoplasmic Ca^2+^ concentration (Zhang *et al*., 2007). Interestingly, it seems that apoplastic Ca^2+^ influx plays an indispensable role during stomatal response to darkness (Schwartz *et al*., 1988).

Dynamic reorganisation of actin microfilaments during plant growth and stress response is orchestrated by a plethora of actin‐binding proteins (ABPs) (Hussey *et al*., 2006; Li *et al*., 2015). Several ABPs that mediate stimulus‐elicited actin dynamics in guard cells have been identified (Zhao *et al*., 2011, 2016; Jiang *et al*., 2012; Li *et al*., 2013, 2014). In earlier work we showed that the Arp2/3 complex regulates stomatal response to ABA, CaCl_2_ and darkness (Jiang *et al*., 2012). A later study showed that ABA‐induced actin remodelling is downstream of reactive oxygen species (ROS) generation, and affects intracellular ROS accumulation through forward feedback (Li *et al*., 2014). SCAB1, another plant‐specific ABP, has also been suggested to couple actin dynamics to stomatal responses (Zhao *et al*., 2011). In addition to the aforementioned ABPs that presumably mediate actin polymerisation and bundling, the actin‐severing protein ADF4 also plays an important role in ABA‐induced actin disintegration through the CKL2 kinase (Zhao *et al*., 2016). Although the involvement of ABPs in stomatal movement has been well established, it is not clear how their activity is modulated by environmental signals.

In this study, we characterised an *Arabidopsis* stomatal darkness‐unresponsive mutant *opal5*, which was recovered from a previous genetic screen (Costa *et al*., 2015). We showed that the phenotype of the *opal5* mutant was caused by a mutation in the *PIR1* gene encoding a subunit of the SCAR/WAVE complex that controls actin cytoskeletal dynamics. On this basis, we further demonstrated the functional significance and the regulatory mechanism of actin filament reorganisation in guard cells during dark‐induced stomatal closure.

## Materials and Methods

### Plant materials and growth conditions

The ecotype of *Arabidopsis thaliana* (L.) Heynh. used in this study was Columbia‐0 (Col‐0). *Atpir‐1* (GABI‐Kat 313F03 for At5g18410) and *Atnap‐1* (SALK_038799 for At2g35110) mutant lines were obtained from the *Arabidopsis* Biological Resource Center (ABRC), and homozygosity of each line was verified by PCR using the primers listed in Supporting Information Table S1. The *wrm‐1* and *dis1‐1* mutants were kindly provided by Dr Jie Le (Institute of Botany, Chinese Academy of Sciences, Beijing, China). Plants constitutively expressing green fluorescent protein (GFP)‐tagged *FABD2* were previously described (Voigt *et al*., 2005).

Seeds were surface‐sterilised with 70% (v/v) ethanol, rinsed thoroughly with double distilled H_2_O, stratified at 4°C for 2 d, and germinated on half strength Murashige and Skoog agar (0.8%, w/v) medium supplemented with 1% (w/v) sucrose. Seedlings were grown in media at 22°C under a 10 h : 14 h photoperiod, with an irradiance of 125 μmol m^−2^ s^−1^. Ten‐day‐old seedlings were repotted into peat‐based compost (Klasmann‐Deilmann, Geeste, Germany) and grown under the same conditions until used for experiments.

### Measurements of stomatal aperture

Stomatal movement was studied using epidermal bioassays as described (Jiang *et al*., 2012). Abaxial epidermal strips of fully expanded rosette leaves from 5‐wk‐old plants were peeled and floated on 10 mM MES/KOH buffer (pH 6.2) for 30 min to close stomata. The peels were then transferred to opening buffer (10 mM MES/KOH, 50 mM KCl, pH 6.15) and incubated under white light (125 μmol m^−2^ s^−1^) for 3 h. To study stomatal closure induced by darkness, the light‐treated epidermal peels were transferred to darkness for 3 h. For ABA and CaCl_2_, these treatments were added after 3 h of incubation in the light and then apertures were measured after a further 3 h in the light. Stomatal aperture was measured at the end of each treatment on an inverted microscope (Eclipse E600; Nikon, Tokyo, Japan). To pharmacologically manipulate actin filament disassembly in wild‐type and *opal5* guard cells, latrunculin B (LatB) or cytochalasin D (CytD) (Abcam, Cambridge, MA, USA) dissolved in ethanol was added to the opening buffer to a final concentration of 10 μM and the peels were treated for 15 min before transfer to treatment conditions.

### Phenotyping using infrared thermography

Phenotyping for lower leaf temperature in darkness was carried out according to Costa *et al*. (2015). Thermal imaging was performed using a ThermaCam B20HS camera (FLIR Systems, Wilsonville, OR, USA) equipped with an uncooled 320 × 240 microbolometer array detector in the 7–13 μm spectral band and an SC5000 camera (FLIR Systems) equipped with a Stirling‐cooled 320 × 256 InSb array detector in the 2.5–5.1 μm spectral band. Thermal sensitivity of the two cameras was such that noise‐equivalent differential temperature (NEDT) was below 0.05°C.

### Mapping of the *opal5* mutation using next‐generation sequencing

The *opal5* mutant was backcrossed three times to the parental Col‐0 line and the reselected mutant progeny was crossed with Ler. Genomic DNA of 50 resulting F_2_ plants exhibiting the *opal5* cooler phenotype was pooled and sent for 2× 100 bp sequencing on the Illumina Genome Analyzer IIx platform. Sequence data were quality‐assessed with FastQC (Babraham Institute, Cambridge, UK), and trimmed with Trimmomatic (Bolger *et al*., 2014) to yield 65.4 million paired reads that were subsequently aligned to the *Arabidopsis thaliana* TAIR10 reference genome using Bowtie2 (Langmead & Salzberg, 2012) on very sensitive settings. Variants were subsequently called and the VCF files were generated using SAMtools (Li *et al*., 2009) and BCFtools. The SHOREmap method (Schneeberger *et al*., 2009) was used to remove background Ler variants, filter variants by EMS type (G to A or C to T), allele frequency, read depth and mapping quality, and to map variant density using a sliding window approach. This identified a region enriched in variants on chromosome 5, centred at *c*. 8 Mbp. Single nucleotide polymorphisms (SNPs) in this region that passed filtering were extracted and annotated using SHOREmap annotate with the TAIR10 release gene models, and mutants with no predicted function were discarded. Five genes (At5g22794, At5g23450, At5g19590, At5g25020 and At5g18410) in this region contained an SNP. These SNPs induced two amino acid changes (T to S in At5g22794 and A to T in At5g23450) and a predicted splice site change in At5g18410, with the remaining variants being synonymous or intronic. Allelic T‐DNA insertion lines were ordered from ABRC and only the homozygous line GABI‐Kat 313F03 that is allelic to At5g18410 phenocopied *opal5*.

### Microscopy

Transgenic plants expressing *35S:GFP‐fABD2* (Voigt *et al*., 2005) were crossed with the *opal5* mutant, and the F_3_ plants homozygous at both loci were used to visualize actin filaments in guard cells of mature leaves from 3‐wk‐old seedlings. Detached leaves were incubated on 10 mM MES/KOH buffer (pH 6.2) for 0.5 h and then floated on the opening buffer (10 mM MES/KOH, pH 6.15, and 50 mM KCl) under white light (125 μmol m^−2^ s^−1^) for 3 h. Half of the light‐treated leaves were incubated under darkness for 30 min. The abaxial epidermis of the leaves was used to observe the guard cell actin cytoskeleton on an LSM 710 laser confocal microscope (Zeiss, Oberkochen, Germany) equipped with a ×63 oil‐immersion objective. Images of guard cells were acquired by serial optical sectioning (Z‐stack) at 1 μm intervals. The samples were excited at 488 nm and emission was detected using a 505 to 530 nm band pass filter. To observe leaf trichomes, the first pairs of true leaves of 10‐d‐old seedlings were fixed overnight at 4°C and dehydrated with ethanol. The specimen was then subjected to a critical point drying process, sputter‐coated and examined on an S‐3000N scanning electron microscope (Hitachi, Tokyo, Japan).

### Quantification of bundling of guard‐cell actin filaments

The Z‐stack images of stomatal guard cells expressing *GFP‐fABD2* were used to quantitatively assess bundling of actin filaments. Serial images were projected, and the skewness of the distribution of GFP‐fABD2 fluorescence intensity in the overlaid images was used as an indicator of the extent of filament bundling. Skewness was quantified using the ImageJ software (http://rsb.info.nih.gov/ij/) as previously described (Higaki *et al*., 2010). Images of 100 guard cells (50 stomata) per genotype or treatment were collected and analysed.

### Accession numbers

Sequence data from this article can be found in the *Arabidopsis* Genome Initiative or GenBank/EMBL databases under the following accession numbers: PIR/SRA1/KLK, At5g18410; NAP1, At2g35110; ARP2, At3g27000; ARP3, At1g13180; ARPC2, At1g30825.

### Statistical analyses

All data analyses were performed using the IBM Spss 20.0 software. Statistical significance was assessed by one‐way or two‐way ANOVA (*P *<* *0.05) as indicated.

## Results

### Dark‐induced stomatal closure is prevented in *opal5*

**Figure 1:**
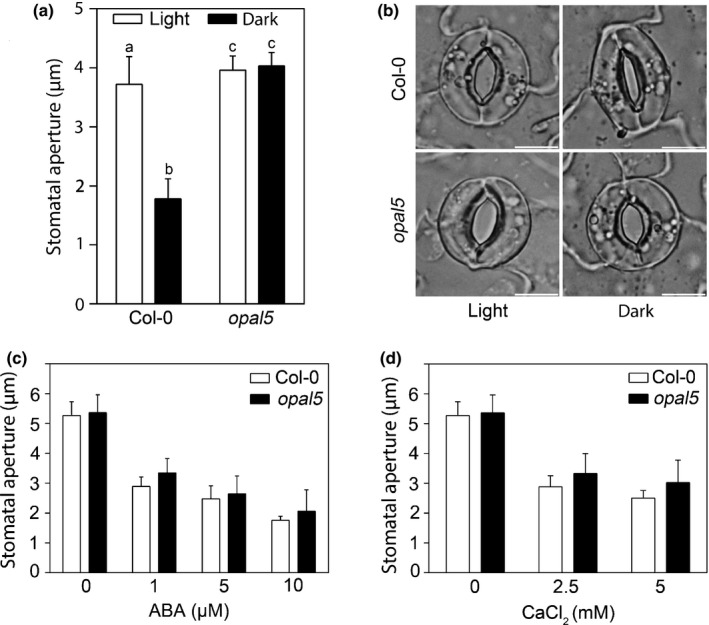
Stomata in the *Arabidopsis opal5* mutant are insensitive to darkness and display wild‐type response to abscisic acid (ABA) and CaCl_2_. (a) Stomatal sensitivity to light–dark transition in wild type Col‐0 and *opal5* mutants. Data are means ± SD (*n *=* *120 stomata per condition, genotype blind analyses). Statistical analyses were performed by one‐way ANOVA, and letters show significant differences at *P *<* *0.05. (b) Representative stomatal images before and after 3 h of dark treatment. Bar, 10 μm. (c) Stomatal bioassays for ABA‐induced closure. (d) Stomatal bioassays for Ca^2+^‐induced closure. Data in (c) and (d) are means of 120 stomatal aperture measurements from three replicates ± SD. Statistical analyses were performed by two‐way ANOVA. *P *>* *0.05 in (c) and (d).

The *opal5* mutant was recovered from a genetic screen for individuals failing to exhibit dark‐induced stomatal closure and is inherited as a single recessive Mendelian locus (Costa *et al*., 2015). To ascertain whether OPAL5 was generally required for stomatal regulation or had a specific role during dark‐induced stomatal closure, we performed leaf epidermal bioassays to analyse stomatal response to extrinsic stimuli in both WT and *opal5* plants. Compared with WT, dark‐induced stomatal closure was completely abolished in the *opal5* mutant (Fig. 1a,b). By contrast, *opal5* showed a WT response to ABA (Fig. 1c) (Costa *et al*., 2015) or CaCl_2_ (Fig. 1d). These observations suggest that OPAL5‐mediated dark‐induced closure is at least partly independent from ABA or CaCl_2_ signalling.

### *opal5* is an allele of the *PIR1* gene that encodes a subunit of the SCAR/WAVE complex

**Figure 2:**
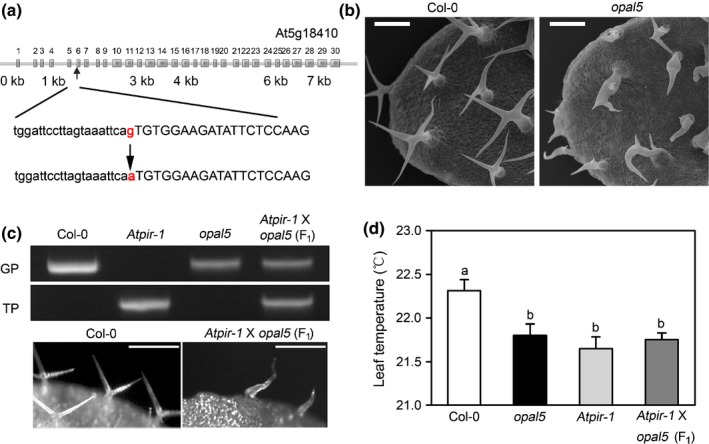
The *opal5* mutation is a new allele of the *Arabidopsis PIR1* gene. (a) Mapping of *opal5* using next generation sequencing. The mutation in *opal5* maps to the At5g18410 gene. Exons are presented as boxes and introns are shown as lines. (b) Scanning electron micrograph of leaf trichomes. (c) Molecular characterization and trichome morphology of the F_1_ progeny of the cross between *opal5* and *Atpir‐1*. Upper panel, PCR identification of T‐DNA insertion with T‐DNA‐specific and gene‐specific primer pairs; lower panel, comparison of trichomes in the wild type and F_1_ progeny of the cross between *opal5* and *Atpir‐1*. Images show leaves of 14‐d‐old seedlings. (d) Complementation test of *opal5* with *Atpir‐1*. Col‐0, *Atpir‐1*,* opal5* and the F_1_ progeny of the cross between *opal5* and *Atpir‐1* were placed in the dark for 6 h and their leaf temperature average was measured after this period by infrared thermograph. The F_1_ progeny showed a similar phenotype to the parental lines (one‐way ANOVA,* P *<* *0.01, *n *=* *4), suggesting that the two recessive mutations *opal5* with *Atpir‐1* are allelic. Error bars are ± SD. Bars, 100 μm.

To determine the identity of the gene responsible for the *opal5* phenotype, we used next‐generation sequencing and identified a genomic region enriched in variants on chromosome 5 (Fig. S1). Five genes (At5g18410, At5g19590, At5g22794, At5g23450 and At5g25020) in this region contained an SNP. Further analysis suggested that the At5g18410 gene contains an SNP at the end of its 5th intron, which introduces a G to A nonsense mutation (Fig. 2a). This gene was previously shown to encode the PIR1 subunit of the *Arabidopsis* SCAR/WAVE complex (Li *et al*., 2004). Similar to the other reported mutants of *PIR1* (Li *et al*., 2004), the *opal5* mutant displayed a distorted trichome phenotype (Fig. 2b). To provide genetic evidence confirming that *opal5* was allelic to *PIR1*, we obtained *Atpir‐1*, a previously characterised T‐DNA insertional line of *PIR1*, and crossed *opal5* and *Atpir‐1* mutants. The F_1_ progenies displayed a distorted trichome phenotype (Fig. 2c), suggesting that *opal5* is an allele of *PIR1*. We then performed stomatal bioassay analyses on *Atpir‐1* and demonstrated that dark‐induced stomatal closure was also abrogated in this mutant, while the mutant showed a WT response to ABA or CaCl_2_ (Fig. S2). A genetic complementation test using thermography after a 6‐h dark period showed that the F_1_ progenies have the same temperature phenotype as the parental lines (Fig. 2d). From these data we can conclude that both recessive mutations *Atpir‐1* and *opal5* are in the same gene and are allelic. Moreover, the *opal5* and *Atpir‐1* mutants display a similar rosette and inflorescence morphology (Fig. S3).

### Non‐allelic mutants of the SCAR/WAVE and Arp2/3 complexes show impaired dark‐induced stomatal closure

**Figure 3:**
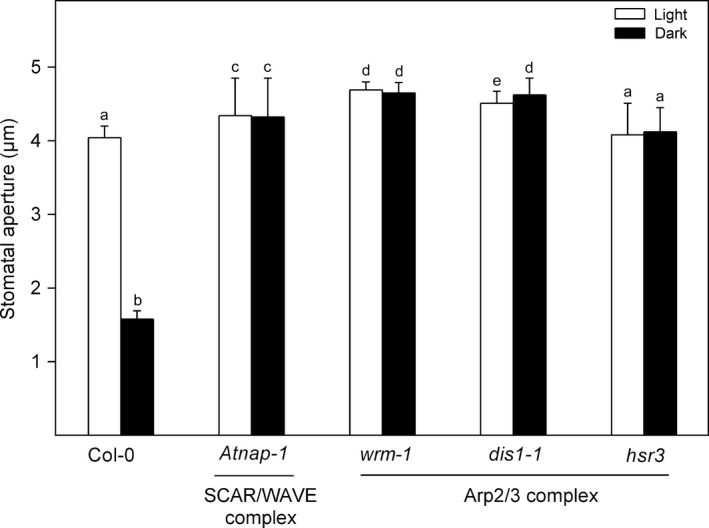
Nonallelic *Arabidopsis* mutants of the SCAR/WAVE and Arp2/3 complexes show a similar stomatal phenotype as the *opal5* mutant. Data are means of 120 stomatal aperture measurements from three replicates ± SD. Statistical analyses were performed by one‐way ANOVA, and letters show significant differences at *P *<* *0.05.

The SCAR/WAVE complex and the downstream Arp2/3 complex form a conserved actin regulatory module in most plants (Deeks *et al*., 2004; Yanagisawa *et al*., 2013), and each is composed of multiple functional subunits (Li *et al*., 2003). To determine whether mutations in other subunits caused defects in stomatal behaviour in the dark, we performed stomatal bioassays on a subset of mutants of the two protein complexes that were previously shown to display aberrant actin cytoskeletons. Specifically, *Atnap‐1* carries a defective NAP1 subunit of the SCAR/WAVE complex (Li *et al*., 2004), while the *wrm‐1*,* dis1‐1* and *hsr3* mutations introduce lesions in the ARP2, ARP3 and ARPC2 subunits of the Arp2/3 complex, respectively (Le *et al*., 2003; Jiang *et al*., 2012). Like the *opal5* mutant, stomata of all the tested non‐allelic mutants of the SCAR/WAVE and the Arp2/3 complexes showed reduced sensitivity to darkness (Fig. 3), indicating that the SCAR/WAVE‐Arp2/3 module mediates stomatal response to darkness.

### Dark‐induced actin reconfiguration is inhibited in *opal5* stomata

**Figure 4:**
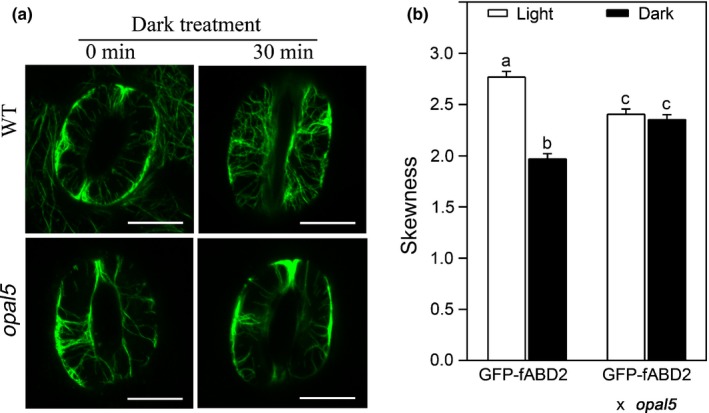
Actin filament remodelling in *Arabidopsis opal5* guard cells shows aberrance in response to dark treatment. (a) Guard cell actin visualized with *GFP‐fABD2* using confocal microscopy. Representative images of wild‐type (WT) and mutant stomata are shown. Bars, 10 μm. (b) The extent of filament bundling (skewness) in stomatal guard cells. Fifty stomata were analysed per genetic background/treatment. Values represent mean ± SE. Statistical analyses were performed by one‐way ANOVA, and letters show significant differences at *P *<* *0.05.

Because the SCAR/WAVE complex plays important roles in plant actin filament patterning (Yanagisawa *et al*., 2013), we examined whether the *opal5* mutation alters dark‐induced actin reorganisation in guard cells. The *opal5* mutants were crossed with plants constitutively expressing the actin fluorescent reporter *GFP‐fABD2* (Voigt *et al*., 2005), and F_3_ plants homozygous at the two loci (Fig. S4) were used for visualization of the actin cytoskeleton. As shown in Fig. 4(a), actin filaments in both WT and *opal5* guard cells were largely bundled and arranged in a radial pattern before dark treatment. A 30‐min dark treatment induced reorganisation of guard‐cell actin filaments into an irregular, mesh‐like arrangement in WT, whilst most actin filaments in *opal5* guard cells remained bundled during the same treatment. To quantify the extent of actin filament bundling in guard cells, skewness was measured before and after dark treatments (Higaki *et al*., 2010). The dark treatment caused a significant decrease in skewness in WT guard cells (Fig. 4b), suggesting that the actin bundles underwent redistribution in response to darkness. By contrast, skewness in *opal5* guard cells remained unchanged after the dark treatment (Fig. 4b).

**Figure 5:**
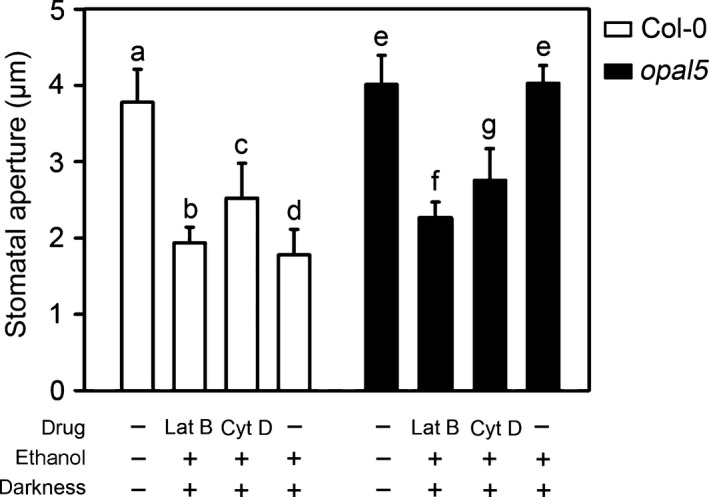
Stomatal insensitivity to darkness in *Arabidopsis opal5* plants is rescued by latrunculin B and cytochalasin D. Freshly prepared abaxial epidermal peels were floated on 10 mM MES and 50 mM KCl (pH 6.15) under 125 μmol m^−2^ s^−1^ light for 2 h and 45 min, then incubated on the same buffer, or transferred to the buffer containing 10 μM latrunculin B (Lat B),10 μM cytochalasin D (Cyt D) or ethanol (dissolvent control) under the same light regime for 15 min. The different batches of peels were subsequently subjected to 0 or 3 h of dark treatment. Stomatal aperture was measured at the end of each dark treatment as indicated. Data are mean ± SD (*n *=* *120 stomata per condition, genotype blind analyses). Statistical analyses were performed by one‐way ANOVA, and letters show significant differences at *P *<* *0.05.

We next investigated whether the stomatal phenotype of *opal5* was a consequence of the apparent absence of dynamic bundle reconfiguration in guard cells. The epidermal peels were treated with either the actin‐monomer sequestering agent LatB or the filamentous actin capping agent CytD before being moved to the dark conditions. The stomatal phenotype in *opal5* was rescued by both LatB and CytD pretreatments while the WT dark response of Col‐0 apertures remained unaffected (Fig. 5). Moreover, the rescued stomatal closure response in *opal5* was not due to the toxic effects of both actin antagonists (Fig. S5). These observations suggest that a reduction in polymerisation releases inhibition from dark‐promoted stomatal closure present in *opal5* guard cells.

## Discussion

Incomplete night‐time stomatal closure has been found across a wide range of plant species, implying evolutionary benefits for plant growth and fitness (Caird *et al*., 2007). Actin filaments in guard cells undergo fast remodelling upon dark treatment, which has been suggested to function as a molecular linker between perception of the dark signal and rapid stomatal responses (Eun & Lee, 1997). In this study, we provide new insights into the dark‐induced closure signalling pathway in guard cells via characterisation of the *opal5* mutant. We showed that the *opal5* mutation specifically disrupts stomatal response to darkness but has no effect on ABA‐ or CaCl_2_‐induced stomatal closure (Fig. 1). After demonstrating that *OPAL5* encodes the PIR subunit of the *Arabidopsis* SCAR/WAVE complex (Fig. 2), we showed that null mutants of the SCAR/WAVE complex and of its downstream Arp2/3 complex fail to exhibit stomatal closure under dark conditions (Fig. 3). This is consistent with the evidence that the SCAR/WAVE complex is the sole activator of the Arp2/3 complex in higher plants (Deeks *et al*., 2004; Yanagisawa *et al*., 2013). By comparing and pharmacologically manipulating actin behaviours in both WT and *opal5* plants (Figs 4, 5), we demonstrated that disorganisation of guard‐cell actin filaments mediated by the SCAR/WAVE complex plays an indispensable role in stomatal response to darkness.

Evidence supporting the hierarchical model between the SCAR/WAVE and the Arp2/3 complexes mainly comes from the results of genetic epistasis tests on subunits of the two complexes (Deeks *et al*., 2004). Our epidermal bioassay results are in agreement with the scenario in which the dark signal is transduced to the actin cytoskeleton via the SCAR/WAVE‐Arp2/3 module (Figs 1a,b, 3). However, although our previous studies revealed the functional significance of the Arp2/3 complex in stomatal response to ABA and CaCl_2_ (Jiang *et al*., 2012), we found that *opal5* mutants have WT sensitivities to those exogenous stimuli (Fig. 1c,d). There are two possible explanations that might explain this discrepancy. The first is that CaCl_2_ and ABA‐mediated stomatal closure does not involve the SCAR/WAVE complex and works through a hitherto unidentified activator of the Arp2/3 complex. An alternative explanation is that the discrepancy is caused by residual SCAR/WAVE activity in the *opal5* mutant, which is sufficient for the transduction of ABA or CaCl_2_ closure‐inducing signals. In support of the former possibility, several studies have shown differential effects between Arp2/3 complex and SCAR/WAVE complex mutants. For example, when compared with Arp2/3 complex mutants, some SCAR/WAVE complex mutants display a less severe reduction in trichome branch length, whilst trichomes of all the mutants display, to a similar extent, abnormally expanded interbranch zones (Basu *et al*., 2005; Le *et al*., 2006; Zhang *et al*., 2008).

**Figure 6:**
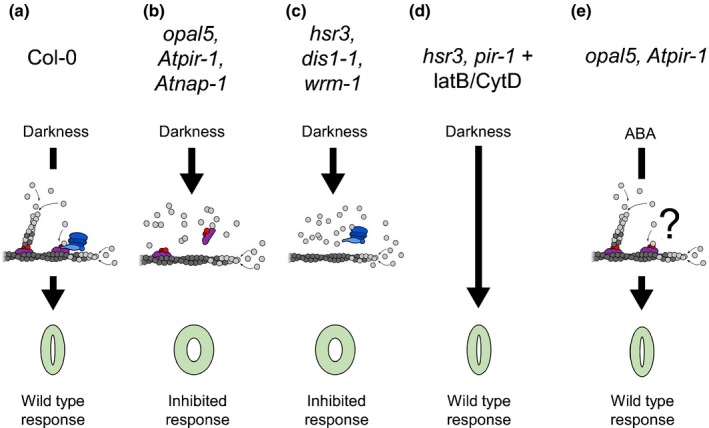
A graphical representation of the *opal5* closure phenotype and related phenotypes in the context of SCAR/WAVE complex and Arp2/3 complex activity. Wild‐type SCAR/WAVE and Arp2/3 complexes (a; represented in blue and magenta/red, respectively) co‐operate to initiate branch structures on older, ADP‐complexed filaments (dark grey). Genetic disruption of the SCAR/WAVE complex (b) or the Arp2/3 complex (c) blocks dark‐induced closure. Note that ATP‐actin monomers (light grey) that would otherwise utilise branched filament ends are now available for polymerisation through other processes. The block to closure is relieved by antagonists of actin dynamics (d).The darkness‐induced phenotype is compatible with the canonical model of plant Arp2/3 complex activation via the SCAR/WAVE complex. Abscisic acid (ABA)‐induced closure is not inhibited by SCAR/WAVE complex disruption (e) but is sensitive to mutants of the Arp2/3 complex (Jiang *et al*., 2012). This does not fully concur with the established role of the SCAR/WAVE complex. It may suggest alternative factors are capable of activating the Arp2/3 complex under the experimental conditions of the ABA assay or that partial activity from incomplete SCAR/WAVE complexes is sufficient for closure under these conditions.

The combined evidence from our studies and those of other actin binding proteins (Zhao *et al*., 2016) supports a model in which dynamic and dendritic/branched networks play a key role in effecting guard cell closure. In this scenario the reconfiguration of the fABD2‐labelled bundled network that occurs during closure (Fig. 4a) is a downstream consequence of the upstream function of actin arrays supported by the Arp2/3 complex and its activators (Fig. 6). This is distinct from a direct biochemical action on bundle reconfiguration.

Single‐filament branching in actin networks is challenging to visualize even at the resolutions of the electron microscope. In animal and fungal cells, enriched labelling of F‐actin highlights sites of high Arp2/3 complex activity such as lamellipodia and endocytic patches. Analogous extremes of dendritic F‐actin enrichment are not widely observed in higher plant cells, suggesting that these structures are either prohibitively dynamic or rarified amongst a brighter population of stable bundled cables. The apical tip of developing trichome branches is an exception (Yanagisawa *et al*., 2015) and demonstrates that relatively isolated and nebulous arrays of branch‐rich actin can have significant impacts on cell morphogenesis. Our genetic data therefore probably indicate the action of a distinct subset of dynamic F‐actin arrays that will prove to be a test for live‐cell imaging approaches.

A connection has been established recently between SCAR complex membrane association and changes in cell pressure (Wang *et al*., 2016). This offers a potential explanation for the functional relevance of branched F‐actin. It has long been established that the response of membrane‐integrated stress‐responsive calcium channels requires a dynamic actin cytoskeleton (Wang *et al*., 2004; Zhang *et al*., 2007). It seems increasingly likely that specific actin network configurations contribute to the coupling of physical and physiological factors during guard cell closure dynamics, and our work presents the Arp2/3 complex and its regulators as candidate molecular players in this process.

Another function that might be exerted by dynamic actin during stomatal movement is regulation of membrane recycling (Zhao *et al*., 2010; Bou Daher & Geitmann, 2011), which would affect subcellular localization or trafficking of ion channels/transporters (Sutter *et al*., 2007; Li *et al*., 2013). It is also noteworthy that, besides the critical roles of actin dynamics in ABA‐promoted stomatal closure, the stability of actin filaments helps to maintain stomata in the closed state (Zhao *et al*., 2016). Additional work will be required to ascertain whether sustained stomatal closure at night is controlled by a similar mechanism.

Overall, our work identified an ABP‐dependent signalling pathway in guard cells that is required for the dark‐induced stomatal closure (summarised in Fig. 6). Recent work on grapevine had shed light on improvement of water use efficiency by manipulating night‐time transpiration (Coupel‐Ledru *et al*., 2016). In this context our work identifies a possible target for manipulation in order to control water use by plants.

## Author contributions

J‐C.I., Z.X., J.M.C., F.M. and X.O. performed the experiments. T.B. carried out bioinformatics analysis. J‐C.I., K.J., M.J.D., B.G. and A.M.H. designed the experiments, interpreted the data and wrote the paper.

## Supporting information

Please note: Wiley Blackwell are not responsible for the content or functionality of any Supporting Information supplied by the authors. Any queries (other than missing material) should be directed to the *New Phytologist* Central Office.

**Fig. S1** Allele frequency of filtered SNPs in chromosome 5.
**Fig. S2** Stomatal aperture of abaxial leaf epidermis.
**Fig. S3** Comparison of morphology of wild‐type and mutant plants.
**Fig. S4** Identification of F_3_ plants homozygous at both the *opal5* mutation and the *GFP‐fABD2* insertion loci.
**Fig. S5** Effects of latrunculin B or cytochalasin D on the viability of guard cells in abaxial leaf epidermis.
**Table S1** Primers used in this study
