# Supplementary material for: Actin filament reorganisation controlled by the SCAR/WAVE complex mediates stomatal response to darkness
Source: New Phytol. 2017 Jun 21;215(3):1059–67. doi: 10.1111/nph.14655 (PMC5519931; doi:10.1111/nph.14655)
Supplement: Supplementary file 1 — Fig. S1 Allele frequency of filtered SNPs in chromosome 5. Fig. S2 Stomatal aperture of abaxial leaf epidermis. Fig. S3 Comparison of morphology of wild‐type and mutant plants. Fig. S4 Identification of F3 plants homozygous at both the opal5 mutation and the GFP‐fABD2 insertion loci. Fig. S5 Effects of latrunculin B or cytochalasin D on the viability of guard cells in abaxial leaf epidermis. Table S1 Primers used in this study [file NPH-215-1059-s001.pdf]

***New Phytologist* Supporting Information**

Article title: Actin Filament Reorganisation Controlled by the SCAR/WAVE Complex Mediates Stomatal Response to Darkness

Authors: Jean-Charles Isner[1], Zaoxu Xu[2], Joaquim Miguel Costa[3], Fabien Monnet[3], Thomas Batstone[1], Xiaobin Ou[2], Michael J. Deeks[4], Bernard Genty[3], Kun Jiang[2]*, and Alistair M. Hetherington[1*]

Article acceptance date: 25 April 2017

The following Supporting Information is available for this article:

Fig. S1 Allele frequency of filtered SNPs in the five chromosomes. The frequency is plotted with a sliding window approach in blue. The candidate region for SNP analysis is the peak centered on 8 Mbp at chromosome 5

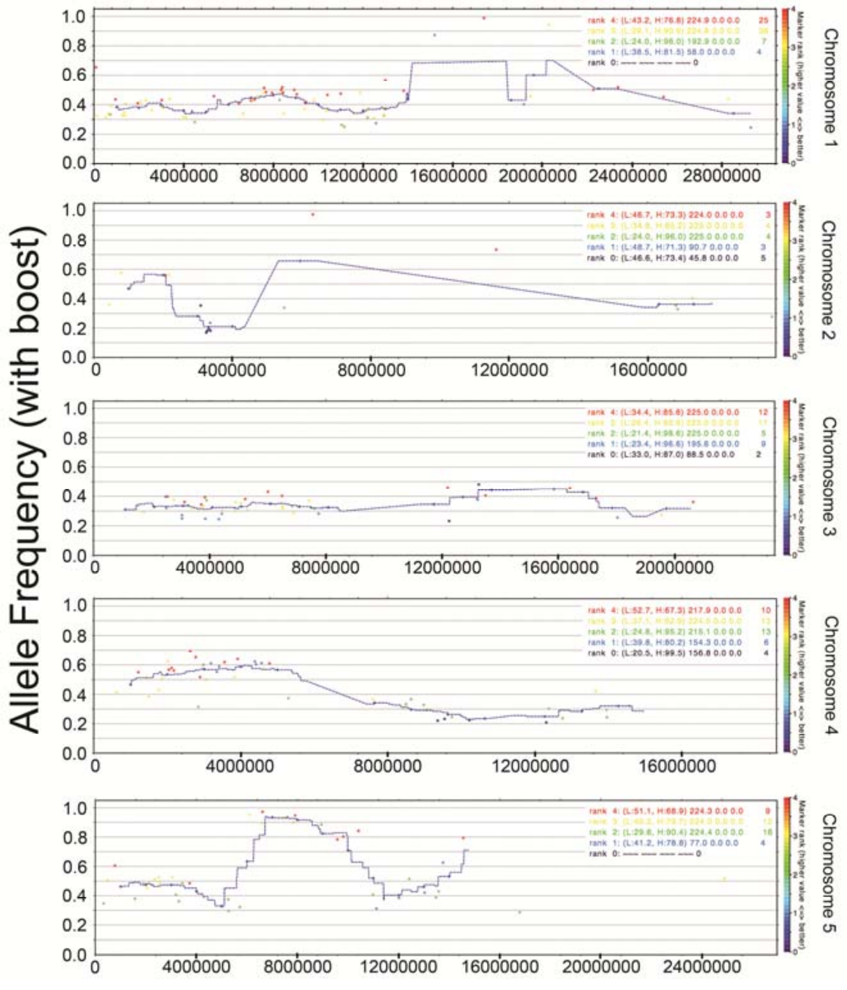

Fig. S2 Stomatal aperture of abaxial leaf epidermis. Stomatal bioassays were performed as described in Figure 1. Data are mean ± SD (n=120 stomata per condition, genotype blind analyses). Statistical analyses were performed by one-way (panel A) or two-way (panels B and C) ANOVA. Letters in (A) show significant differences at $P<0.05$. $P>0.05$ in (B) and (C)

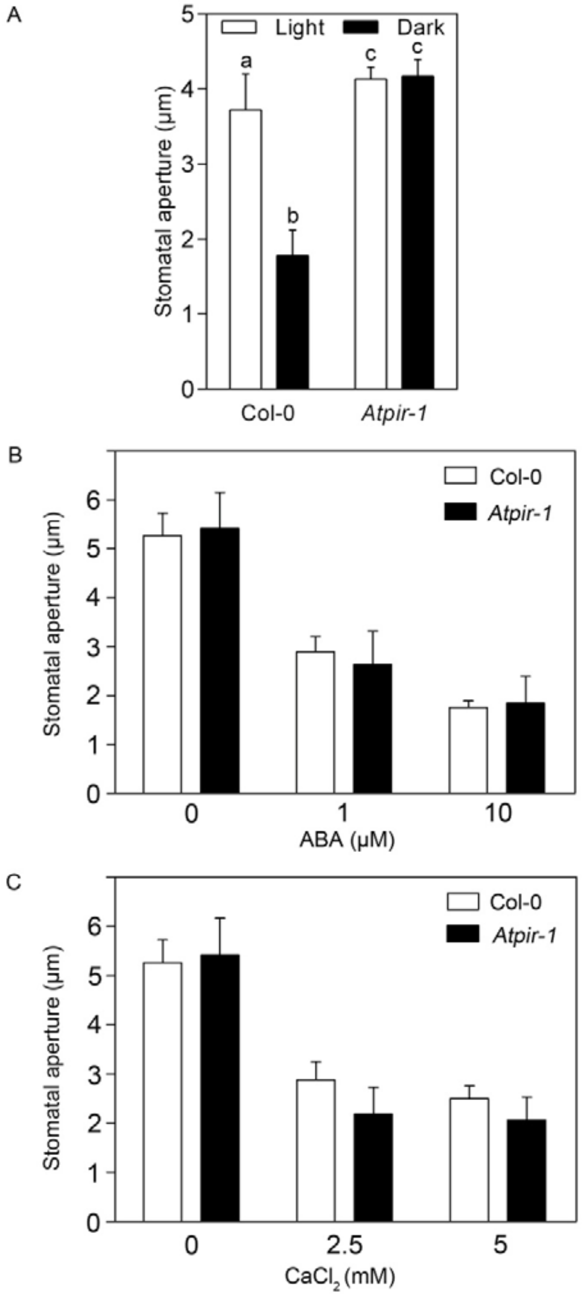

Fig. S3 Comparison of Morphology of Wild-type and Mutant Plants. (A) Mature Col-0, *Atpir-1*, and *opal5* plants. Note inflorescence stems of *Atpir-1*and *opal5* are shorter and display an extended growth. Bar = 5 cm (B) Rosettes of 5-week-old Col-0, *Atpir-1*, and *opal5* plants. Mutants of the *PIR1* gene have paler green rosette leaves. Bar = 5 cm

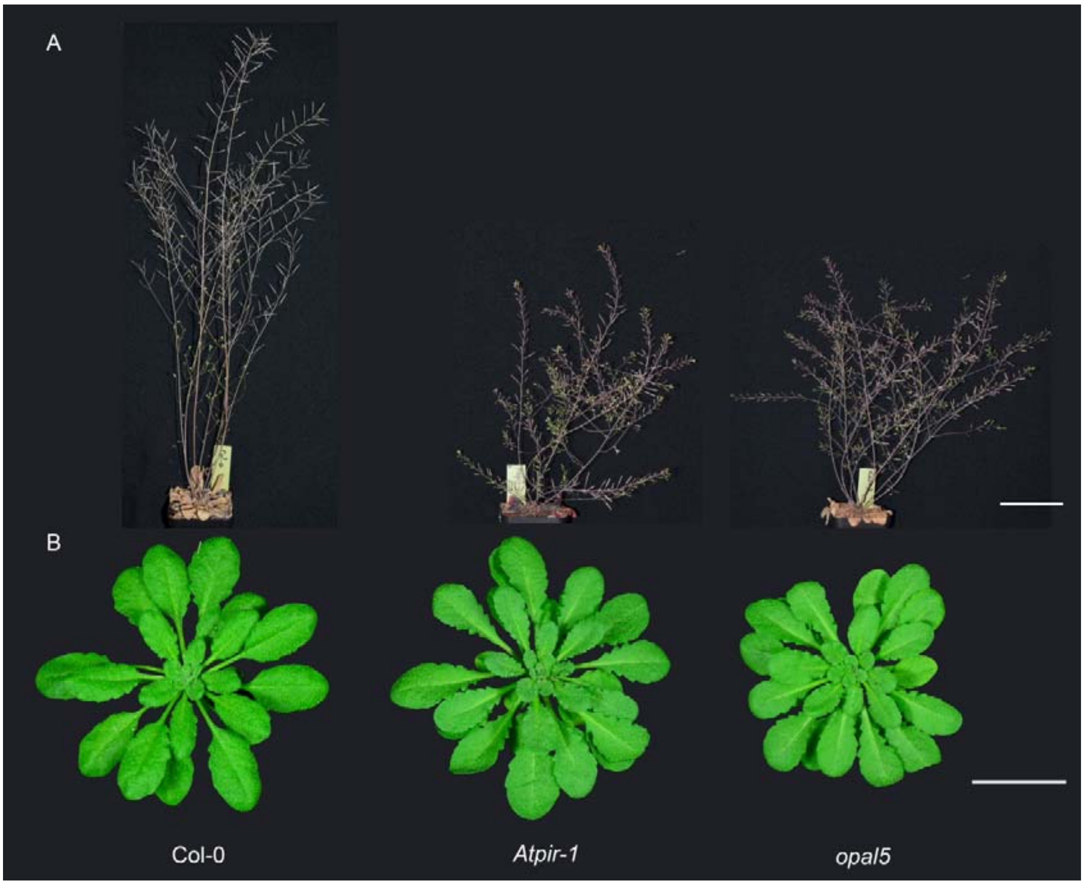

Fig. S4 Identification of F3 Plants Homozygous at Both the *opal5* Mutation and the *GFP-fABD2* Insertion Loci. (A) F1 seedlings of the cross between *opal5*and *35S:GFP-fABD2* show strong GFP fluorescence in the root tip area. Bar = 100μm (B) BASTA (glufosinate ammonium, 25 mg/L) resistance selection of plants. F3 seedlings showing no segregation in BASTA resistance are homozygous for the *GFP-fABD2* insertion.*opal5*: negative selection control;*35S:GFP-fABD2*: positive selection control

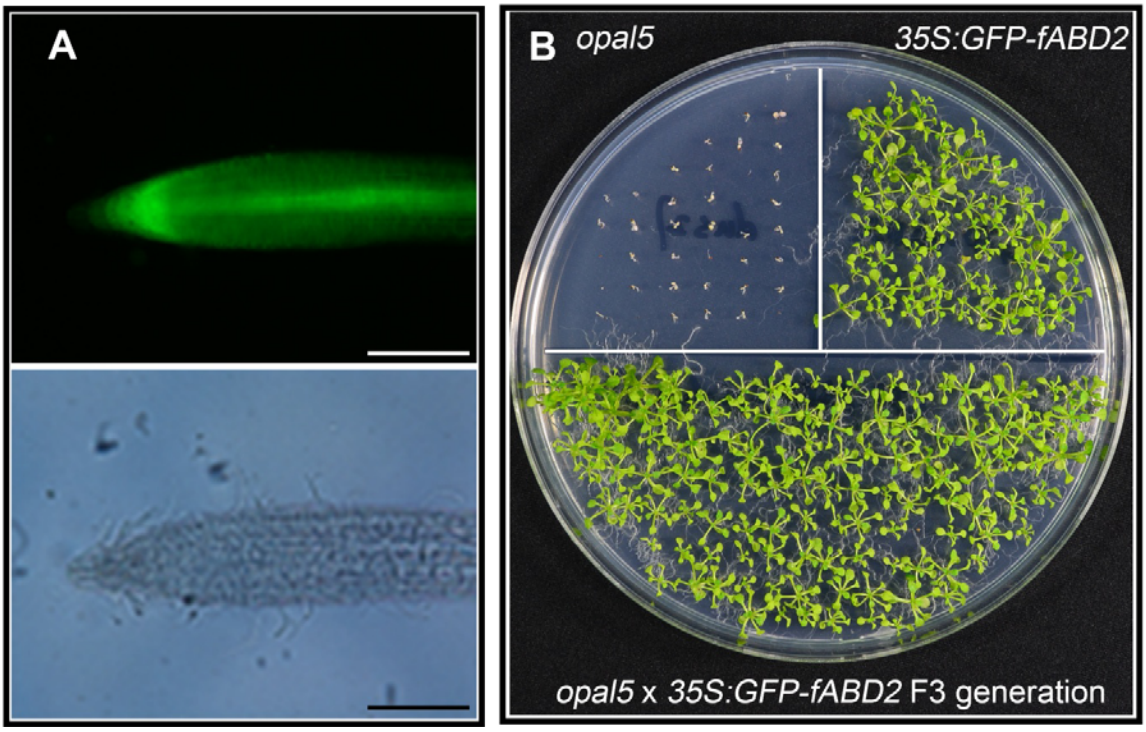

Fig. S5 Effects of Latrunculin B or Cytochalasin D on the Viability of Guard Cells in Abaxial Leaf Epidermis. (A) Fluorescein diacetate (FDA) was used to assay viability of guard cells in abaxial leaf epidermis of wildtype plants. The peels were treated with 10 µM latrunculin B (Lat B) or cytochalasin D (Cyt D) as described in Figure 5. FDA fluorescence was visualized with a Nikon Eclipse Ni-U fluorescence microscope before and after 3 h of dark treatment. The experiment was replicated three times and 2-3 peels were checked in each replicate. Representative bright filed (left) and fluorescent (right) images were shown for each experimental condition. Bar = 100 µm (B) Quantitation of the data from (A). Over 90% of guard cells were alive under all the experimental conditions. Error bars, ± SD (C) Stomatal bioassays for light-promoted opening. Epidermal peels of wildtype leaves were incubated under light and then treated with 10 µM latrunculin B (Lat B) or cytochalasin D (Cyt D) as described in Figure 5. The drug-treated peels were kept under the same light regime, and stomatal aperture was measured at 1-h intervals over a 3-h experimental period. Data are mean ± SD (n=120 stomata per condition, genotype blind analyses).

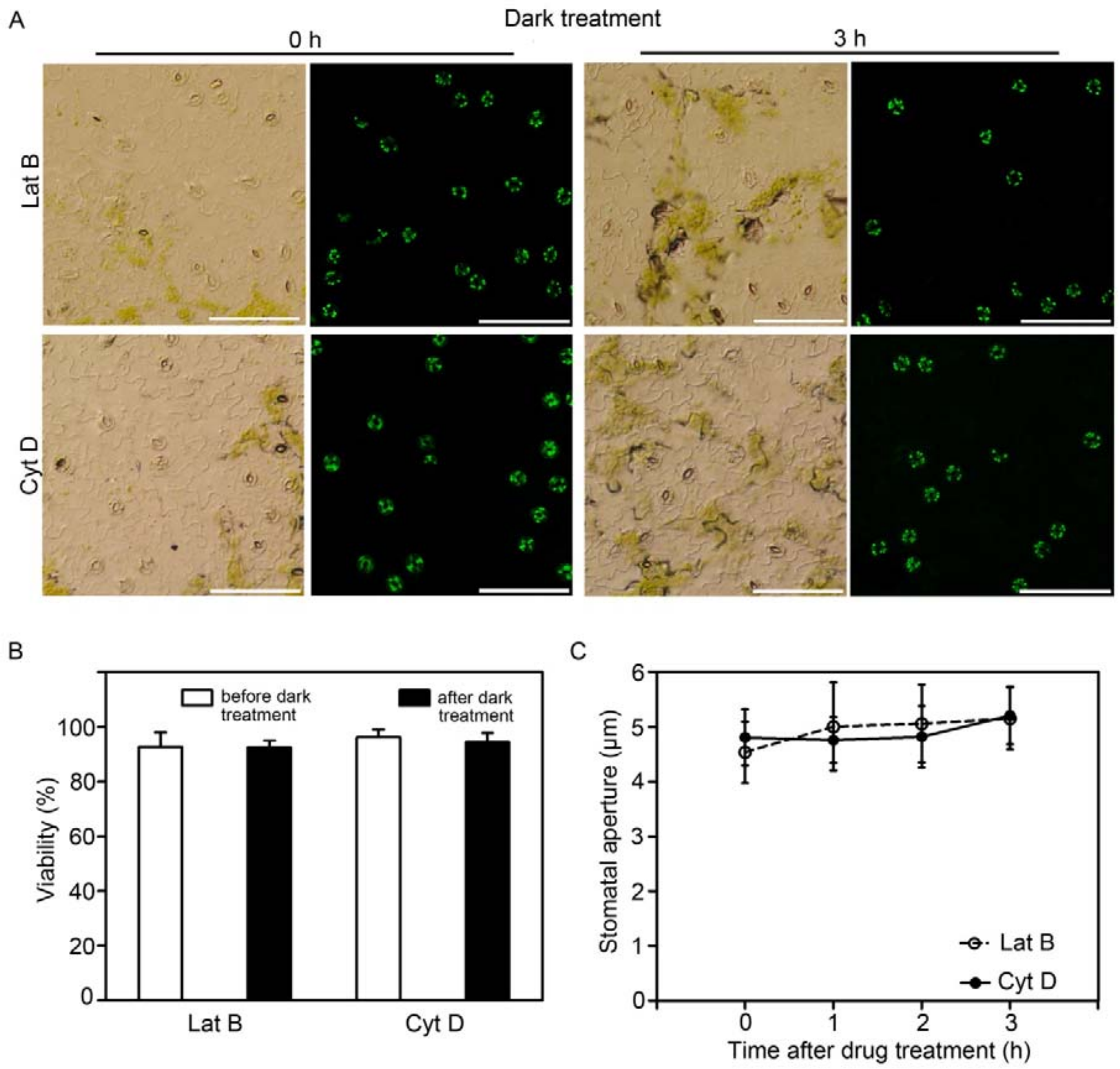

A
Dark treatment
0 h
3 h
Lat B
Cyt D
B
Viability (%)
before dark treatment
after dark treatment
Lat B
Cyt D
C
Stomatal aperture (μm)
Lat B
Cyt D
Time after drug treatment (h)

TableS1 Primers Used in this study

Primer name	Sequence (5’-3’)	Purpose
LBb1.3	ATTTTGCCGATTTCGGAAC	Genotyping
GABI_o8409	ATATTGACCATCATACTCATTGC	Genotyping
pir-1_LP	AGAAGGAAAAGAGATGGCGTC	Genotyping
pir-1_RP	AGGGGAAGGCATTATCAAATG	Genotyping
nap-1_LP	AGGTTTCGAGAGAGACTTGCC	Genotyping
nap-1_RP	GAGAATGGGGTGCTCTTCTTC	Genotyping
